# Genotype by Environment Interactions in Gene Regulation Underlie the Response to Soil Drying in the Model Grass *Brachypodium distachyon*

**DOI:** 10.1093/molbev/msaf218

**Published:** 2025-09-11

**Authors:** Jie Yun, Angela C Burnett, Alistair Rogers, David L Des Marais

**Affiliations:** Civil & Environmental Engineering, Massachusetts Institute of Technology, 15 Vassar St., Cambridge, MA 02139, USA; Environmental & Climate Sciences Department, Brookhaven National Laboratory, P.O. Box 5000, Upton, NY 11973, USA; Environmental & Climate Sciences Department, Brookhaven National Laboratory, P.O. Box 5000, Upton, NY 11973, USA; Civil & Environmental Engineering, Massachusetts Institute of Technology, 15 Vassar St., Cambridge, MA 02139, USA

**Keywords:** G × E, Brachypodium, gene regulation, drought response, causal inference, gene co-expression network

## Abstract

Gene expression is a quantitative trait under the control of genetic and environmental factors and their interaction, so-called genotype and environment (G × E). Understanding the mechanisms driving G × E is fundamental for ensuring stable crop performance across environments and for predicting the response of natural populations to climate change. Gene expression is regulated through complex molecular networks, yet the interactions between genotype and environment in gene regulation are rarely considered, particularly at the genome scale. Current frameworks and experimental designs often lack power to explicitly test network rewiring or to systematically compare regulatory networks. Here, we leverage a highly replicated RNA-sequencing dataset to model genome-scale gene expression variation between two natural accessions of the model grass *Brachypodium distachyon* and their response to soil drying. We first identified genotypic, environmental, and G × E effects on physiological, metabolic, and gene expression traits. We identify patterns of conservation—or variation—in gene coexpression networks and link these coexpression features to physiological traits. We further develop predictions of gene–gene interactions using causal inference and screen for interactions specific to—or with higher affinity in—a single genotype, treatment, or their interaction, G × E. Our analyses identify variation in candidate gene regulatory networks that may shape the evolution of environmental response in *B. distachyon*. We highlight the environmentally dependent regulatory control of several metabolic traits shown previously to play a role in drought acclimation. The framework presented here provides a scalable approach for more complex comparisons, particularly with the growing availability of large datasets from technologies such as single-cell transcriptomics.

## Introduction

Gene expression represents a unique class of quantitative, complex traits. Like all quantitative traits, the transcript abundance of an individual gene can take a wide range of values, can show variation in those values among samples from a population, and may be controlled by a number of intrinsic and extrinsic factors (Wittkopp and Kalay 2012; Hill et al. 2020; Huang et al. 2020; Veltsos and Kelly 2024). And, because gene expression is a component of many quantitative traits such as morphology, life history, behavior, and physiology (National Academies of Sciences 2020), using tools from quantitative genetics to study gene expression may provide insight into the molecular control of these higher-order traits (Rockman 2008).

Variation in a quantitative trait can be modeled as a function of genetic differences among sampled individuals, of the effect of the environment on those individuals (i.e. phenotypic plasticity), and of the interaction between genotype and environment, G × E (Des Marais et al. 2013). Expression quantitative trait locus (eQTL) studies identify genetic loci that are associated with differences in the abundance of transcripts among natural accessions of plants (Lowry et al. 2013; Josephs et al. 2015, 2020; Lovell et al. 2018); these genetic variants describe heritable, constitutive differences in expression among genotypes. Other studies demonstrate the strong effects that differences in environment—either field-based or imposed as treatment in controlled conditions—can have on the expression of single genes or suites of genes, e.g. (Reynoso et al. 2019; Varoquaux et al. 2019; Winkelmüller et al. 2021; Wu et al. 2021).

Perhaps owing to the important role afforded phenotypic plasticity in plant ecology, evolution, and agriculture (Bradshaw 1965), hypotheses about its molecular basis predate modern genomic science (Smith 1990) and remain central to plant biology (Remold and Lenski 2004; Des Marais and Juenger 2010; Levis and Pfennig 2016; Rispail et al. 2018; Fox et al. 2019; Xiong et al. 2021; Choquette et al. 2023). Many -omics studies are predicated on the hypothesis that regulators of transcriptional activity offer attractive targets to manipulate suites of downstream molecular processes and, ultimately, organism-scale phenotypic plasticity (e.g. as described in (Wei et al. 2022; Zhang et al. 2022)). However, identifying genes that can be manipulated to improve performance in the field while not also incurring a fitness penalty (so-called yield drag) remains challenging (Passioura 2020). The molecular variants driving natural genetic variation in environmental response—G × E—represent a means to study how small genetic changes drive environmentally responsive physiological and developmental traits (Des Marais et al. 2013). Because such genetic variants are naturally occurring and have likely been exposed to natural selection in wild populations, understanding their function in vivo may provide insight into how to design synthetic systems to improve stress tolerance without incurring yield drag. Genetic variants affecting G × E also represent the population-level variation on which natural selection might act to optimize fitness in response to climate change (Nicotra et al. 2010) and are thus of interest for predicting the future of natural plant communities.

G × E in plant gene expression has been reported in previous studies, most often at the level of individual genes or at global scale treating each gene as an independent variable (Des Marais et al. 2012; Shin et al. 2015). Suites of genes are often under common regulatory control (Liu et al. 2019), and, as a result, treating each gene as independent is difficult to justify statistically. Collectively, the interactions between genes at the transcriptional level can be conceptualized as a gene regulation network (GRN) that may drive the flow of information through a cell (Alon 2007; Lachowiec et al. 2016). The common network motifs observed in GRNs imply that some regulators directly regulate or are “upstream” of other genes and may therefore exhibit pleiotropic effects when perturbed. GRNs, and the subnetworks, modules, or communities that comprise them, may be seen as an integrated molecular phenotype that allows for identifying transcriptional associations with physiological, metabolic, or developmental functions, thereby improving biological interpretability (Thompson et al. 2015).

The dependency of gene coexpression and the resulting topology of GRNs on environmental variation is poorly understood (Sun and Dinneny 2018). By extension, we have a limited understanding of how environmentally responding networks evolve and might be successfully manipulated using biotechnology to improve crop resilience. For example, genes exhibiting G × E in their expression might be clustered into modules, as it is hypothesized that environmental pressure results in the evolution of modules such that organismal subsystems can function independently (Wagner et al. 2007). Recent work in *Caenorhabditis elegans* demonstrated that G × E genes are associated with a greater number of trans-acting regulators than are genes showing simple genotypic differential expression (Grishkevich and Yanai 2013). Previously, we showed that the number of genes connected to, and the influence of genes showing G × E in expression, is lower under drought stress but higher under cold stress compared to a random set of genes (Des Marais et al. 2017a), again suggesting that the topology of GRNs could have been shaped by—or possibly shaped the efficacy of—past selection. However, that study, like most studies of environmentally dependent gene expression, assumed that coexpression relationships are invariant across genotypes and environmental treatments, which limits our ability to understand the evolution of complex gene expression phenotypes (Luscombe et al. 2004; Lachowiec et al. 2016).

G × E is widely observed in a large variety of species such as yeast (Smith and Kruglyak 2008), *C. elegans* (Grishkevich et al. 2012), *Drosophila melanogaster (*Huang et al. 2020*)*, and human (Virolainen et al. 2023), and its study plays important roles in fields as diverse as crop breeding and human health. Studying G × E in plants facilitates highly replicated experiments with tightly controlled environments using genetically identical individuals. Here, we use two inbred natural accessions of the model grass *Brachypodium distachyon* (Scholthof et al. 2018) to study G × E in response to soil water deficit. *B. distachyon* is closely related to economically significant grasses such as wheat, barley, and oat (Draper et al. 2001; Scholthof et al. 2018). *B. distachyon* populations are found throughout the Mediterranean where most exhibit a winter annual strategy—germinating in the fall or spring and setting seed before the typically hot, dry summer months (López-Alvarez et al. 2015; Des Marais et al. 2017b). We selected two closely related inbred accessions both originating in northern Iraq (Garvin 2016; Tyler et al. 2016) that are geographically close, have similar developmental trajectories, and exhibit high genomic similarity. The accessions are not identical; however, we previously documented G × E in leaf water and metabolite content in this system (Des Marais et al. 2017b) as well as environmentally dependent QTL (Des Marais et al. 2016). We use highly replicated factorial combinations of G × E, harnessing variation from stochastic variance and microenvironmental heterogeneity among replicates to estimate regulatory interactions (Elowitz et al. 2002; Cortijo et al. 2020). We hypothesize that environmentally responsive, genotypically specific, and genotype by environment interaction in transcript abundance and gene–gene interactions are spatially clustered in the gene regulatory network(s) [GRN{s}] of *B. distachyon*. We first estimate modules of coexpressed genes and correlate these with a suite of physiological traits. Within a subset of certain gene coexpression modules, we then infer causal gene networks as hypotheses about gene regulation, including those that represent condition-specific regulatory interactions. The framework we introduce in this study facilitates comparisons of high-dimensional data to elucidate the structure and function of GRNs at whole-genome scale.

## Material and Methods

### Experimental Growth Conditions and Drought Treatments

The experimental protocol was adapted from a study by Des Marais et al. (2016). Seeds of two accessions of *B. distachyon* (Bd21 and Bd3-1) were placed at 4 °C for 7 d to synchronize seedling germination. Seeds were then planted in Deepot D16H pots (Stuewe&Sons) filled with Profile porous ceramic rooting media (Profile Products, Buffalo Grove, IL). The dry weight (DW) of each pot and its field capacity (FC) for water were recorded. FC was determined by saturating a pot with water and allowing it to drain under gravity overnight and calculating the weight difference from DW; this was used as the basis for the subsequent controlled soil dry down. Plants were grown in growth chambers with 50% humidity, 500 μmol photons m^−2^ s^−1^ light intensity, and 25 °C day/20 °C night (12 h/12 h) cycles. Pots were bottom watered with tap water (pH = 5 to 6) every other day, supplemented with DYNA-GRO GROW Liquid Plant Food 7-9-5 (Dyna-Gro, Richmond, CA, United States) over the first 32 d. Starting on Day 33 after sowing, control plants were watered to 85% FC every night, while dry-down plants were allowed to decrease their soil water content to 50% FC over 7 d (5% decrease each day). The plants were divided into two groups with Group 1 used to characterize physiological, metabolite, and developmental responses along the dry-down process and Group 2 used to measure gene expression and physiology and metabolite content on Day 6.

On Days 0 (85%FC), 2 (75%FC), 4 (65% FC), 5 (60% FC), 6 (55% FC), and 7 (50% FC), nine individuals of each genotype from each treatment (control and drying) were measured for gas exchange at 3 to 5 h after lights-on, PSII maximum efficiency of light-adapted leaves (fv′/fm′) at 5 to 6 h after lights-on, and destructive sampling for leaf hydraulic potential, leaf metabolites, and total plant biomass at 6 to 8 h after lights-on. Preliminary analyses determined that differences in photosynthetic and leaf hydraulic parameters were detectable between control and droughted plants on Days 5 and 6; accordingly, we selected Day 6 for detailed analysis. Forty-eight to 59 replicates of leaf tissue in each genotype and treatment were measured for gas exchange and photosynthesis at 1.5 to 5 h after lights-on on Day 6 and harvested for RNA extraction and metabolite assay between 6 and 7.5 h after lights-on (equivalent to midday). Sampling of individuals was randomized to avoid confounding time-of-day measurement or harvest with genotype or treatment. In total, there are 214 plants (2 treatments × 2 genotypes × 48 to 59 biological replicates).

### Physiological and Metabolic Measurements

To characterize the dry-down process, we measured photosynthesis and gas exchange parameters, leaf hydraulic potential, biomass, and leaf metabolites. Net CO_2_ assimilation rate (A) and stomatal conductance (g_sw_) of the youngest fully expanded leaf of a selected tiller were determined using a Li-6800 (LI-COR Biosciences, Lincoln, NE). The LiCor chamber was set to a flow speed of 200 μmol s^−1^, humidity at 50%, CO_2_ concentration at 400 μmol mol^−1^, fan speed of 10,000 rpm, and photosynthetic photon flux density (PPFD) at 1000 μmol (m^−2^ s^−1^). Leaves were left for 5 min to stabilize in the chamber before each reading. PSII maximum efficiency of light adapted leaves (fv′/fm′) was measured on leaves at noon following manufacturer protocols. Leaf chlorophyll content was estimated by taking the average of two leaves measured with MultispeQ V2.0 (Kuhlgert et al. 2016) prior to lights-on in the growth chamber.

The leaf water potential was measured by the Scholander Pressure Chamber Model 670 (PMS Instrument Company, Albany, OR) on the youngest fully expanded leaf of sampled plants.

Above-ground tissue was excised at soil level with a razor blade. Roots were cleaned with tap water to remove growth media and then dried with tissue paper. Fresh tissue biomass was recorded before samples were dried for 48 h at 80 °C and then weighed for dry biomass.

Tissue for RNA and metabolites was flash frozen in liquid nitrogen, stored at −70 °C, and freeze-dried overnight prior to grinding with bead-beating. A total of 20 mg DW was utilized for the following assays. Metabolites were extracted using sequential ethanol extractions following established protocols (Ainsworth et al. 2007; Burnett et al. 2016). Protein, starch, nitrate, total free amino acids, glucose, fructose, sucrose, and high- and low-degree of polymerization fructans were quantified following (Burnett et al. 2016). Metabolite data are expressed here per gram DW, or as glucose equivalents per DW for carbohydrates. Technical and analytical replicates were run for all assays.

### Statistical Analyses

To determine the effects of genotype, treatment, and time on physiological and metabolic responses, most traits collected during dry-down (supplementary data set S1, Supplementary Material online) were fitted into linear mixed models using lm and lmer in the lme4 package (Bates et al. 2015) in R (R Core Team 2022). Glucose and fructose were fitted with generalized linear mixed models with Gamma distribution in glm and glmer (Bates et al. 2015), with family = Gamma(link = “inverse”). Accession (Bd21 and Bd3-1), treatment (control and drought), harvest day (Days 0, 2, 4, 5, 6, and 7), and their interactions were modeled as fixed factors and freeze dry session (four levels) and assay batches (five levels) for metabolites were modeled as random factors. Model selection was first performed on the fixed effects of each model, based on maximum likelihood Akaike information criterion (AIC) using the dredge function in the MuMIn package (Bartoń 2020). Random-effects factors of each model were based on maximum likelihood of models using the anova function in the core R suite. Models are shown in supplementary table S1, Supplementary Material online. Throughout the dry-down process, pairwise significances between treatments for each accession on each harvesting day (supplementary figs. S1 and S2, Supplementary Material online) was estimated with a *t*-test (or *Z*-test for glm and glmer), adjusted by Tukey using the emmeans function in the emmeans package (Lenth et al. 2020) in R based on the model selected. Outliers were removed before model fitting. The results were visualized using ggplot2 and ggpubr (Wickham 2016; Kassambara 2020).

**Fig. 1.**
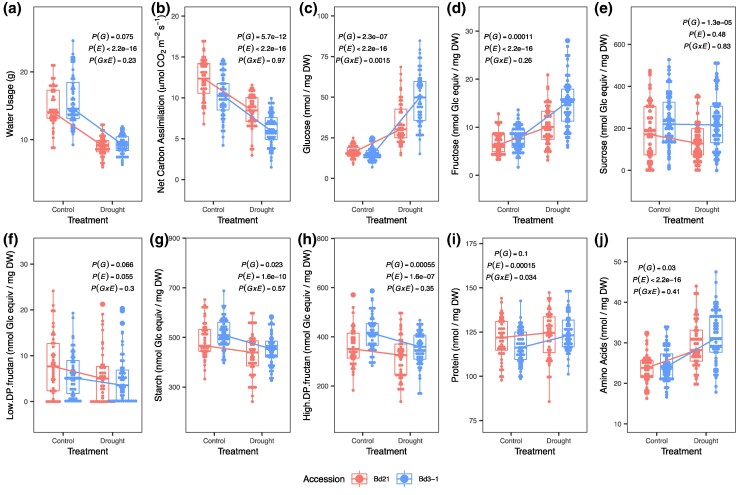
Leaf blade traits of Bd21 and Bd3-1 on Day 6. The *P*-values of effect terms were determined using (generalized) linear (mixed) models, by removing each term and performing likelihood ratio test (anova and Chisq test, with maximum likelihood mode if a model includes random effects). DW, dry weight of tissue; Glc Equiv, glucose equivalents. *N* = 48 to 59 for each combination of accession and treatment.

Similarly, leaf trait data collected on Day 6 (supplementary data set S2, Supplementary Material online) were fit with (generalized) linear mixed models using lm, lmer, glm, or glmer, as appropriate, with fixed factors of accession, treatment, their interaction, and random factors of growth batch, freeze dry sessions, and assay batch for metabolites (Fig. 1). The *P*-values shown are based on likelihood ratio test (Chisq test) of dropping each term using anova function in the core R suite.

### Transcriptome Sampling and Processing

Leaf tissue flash frozen on liquid nitrogen was ground using an MP FastPrep-24 Classic bead beating grinder with CoolPrep 24 × 2 mL Adapter (MP Biomedicals) loaded with dry ice. Approximately 100 mg of tissue was used for RNA extraction using Spectrum Plant Total RNA Kit (Sigma-Aldrich, USA) according to the manufacturer instructions, followed by DNase treatment (On-Column DNase I Digestion Set, Sigma-Aldrich, USA). The quality and quantity of the RNA samples were determined both by absorbance measurements (Nanodrop 1000, NanoDrop Technologies) and with a Tape Station (4200 TapeStation System, Agilent). A total of 25 ng of RNA were processed for High-Throughput 3′ Digital Gene Expression (Soumillon et al. 2014; Xiong et al. 2017) by the MIT BioMicro Center, pooled, and then sequenced across three flowcells on an Illumina Novaseq (NovaSeq6000 Sequencing System, Illumina) generating, on average, 3.9 ± 1.3 million raw reads per sample.

Raw sequence reads were trimmed by Trimmomatic (Bolger et al. 2014) and Cutadapt (Martin 2011) to remove adapters and poly(A) tails. All reads were mapped to the *B. distachyon* Bd21 primary transcriptome sequence (Bdistachyon_556_v3.2.transcript_primaryTranscriptOnly, downloaded from Phytozome) and the annotated sequence of the whole Bd21 plastome, which is available at GenBank (LT558597 accession). Read alignments from Bowtie2 (Langmead and Salzberg 2012) were corrected by RNA-Seq by Expectation-Maximization (RSEM) (Li and Dewey 2011) to account for nonuniquely aligned reads. Bowtie2 alignment was done with seedlength -L 25 and mismatch allowance -n 2, which allowed mismatches of Bd3-1 read alignments and –estimate-rspd, which specified that the read position distributions were highly 5′- or 3′-biased (Li and Dewey 2011). Reads with the same unique molecular identifier and aligned to the same genes were removed to reduce PCR biases during library generation (Soumillon et al. 2014; Xiong et al. 2017) prior to adjustment in RSEM. RNASeq reads from Bd21 and Bd3-1 mapped to the Bd21 references at 77.0 ± 1.7% and 78.1 ± 1.8%, respectively, suggesting no accession effect on mapping. Reads that did not map to the genome during this first round were mapped onto ribosomal RNA, after which the total mapping rates increased to ∼96%. The overall downstream gene expression analysis framework is summarized in supplementary fig. S3, Supplementary Material online.

### Differential Gene Expression

**Fig. 2.**
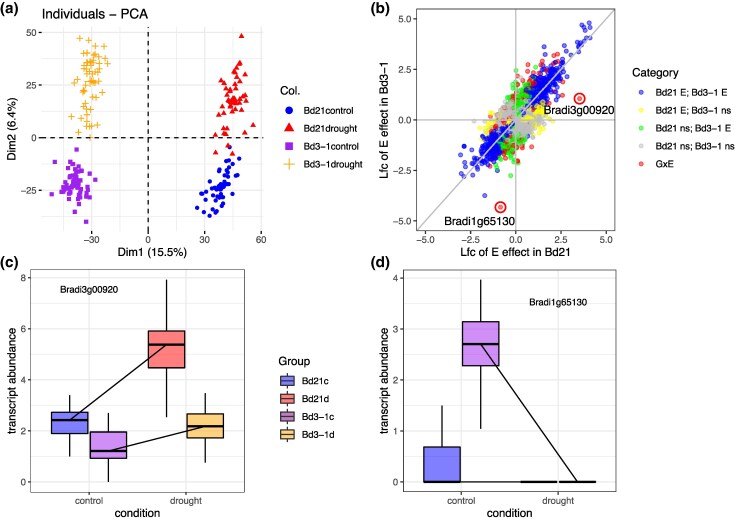
Gene expression diversity. a) PCA shows clustering of RNASequencing library by accession and environmental treatment. b) Factors affecting the transcript abundance of individual genes. Each point represents a gene model in the *B. distachyon* Bd21 genome. Lfc = log-fold change of transcript abundance in response to soil drying. Colors indicate the significance of treatment effect on Bd21 and Bd3-1 samples; genes with a significant G × E effect are highlighted in red and the two genes plotted in c and d) are circled. Boxplots show two genes with large G × E effect: c) Bradi3g00920 and d) Bradi1g65130, gene expression reaction norm based on log2(norm_count + 1).

To determine the effects of genotype and treatment on variance in transcript abundance, RNASeq reads were normalized using relative log expression normalization implemented by DESeq2 (Love et al. 2014). These normalized data with log_2_ (Count + 1) transformation were used for downstream analyses; genes with >0 counts in more than 10 samples were kept. The grouping was visualized with principal component analysis (PCA) using factoextra package in R (Kassambara and Mundt 2020). DESeq2 fit the transcript count of each gene into the generalized linear model Transcript Count ∼ Genotype + Treatment (+ Genotype * Treatment) or Transcript Count ∼ Treatment, stratified by genotype or Transcript Count ∼ genotype, stratified by treatment assuming a negative binomial distribution. Results in Fig. 2 were visualized with ggplot in R (Wickham 2016; Kassambara 2020).

### Identifying Patterns of Variation in Clusters of Gene–Gene Interactions

The preceding analyses sought to partition variance components for individual genes, independent of any latent variables that could explain their relationship to broader patterns of coexpression across the transcriptome. We next identified sources of variation in gene coexpression modules and addressed the possible cause(s) of this variation. For this and following analyses, we removed transcripts exhibiting low variance (<0.1) among replicates in each library type; in total, 16,207 genes were included. Coexpression modules in each library type (Bd21 control, Bd21 drought, Bd3-1 control, and Bd3-1 drought) were generated using Weighted Gene Co-expression Network Analysis (WGCNA) (Langfelder and Horvath 2008). For WGCNA, the starting correlation matrix was unsigned, as both negative and positive correlations are of interest in this study. Weight power = 5 was added to the correlation matrix to achieve a scale-free adjacency matrix. The topology similarity matrices in the Bd21c, Bd3-1d, and Bd3-1c datasets were scaled to have a similar distribution to Bd21d, which we chose arbitrarily as the reference library type. Dendrograms were generated from topology similarity matrices, and modules were generated by cutting at two parameter settings: (i) moderate size modules, used for most analysis below, with high dissimilarity (height = NULL(0.99) and a dynamic hybrid algorithm (minClusterSize = 30, deepsplit = 2, pamStage = FALSE, mergeheight = 0.25), and (ii) conservative (smaller) modules, used for subsequent causal inference analyses, with cutHeight = 0.984, minClusterSize = 15, deepSplit = 3, pamStage = FALSE, and mergeheight = 0.25. The two different settings were used to better understand the effect of threshold choice within each library type. In brief, our WGCN analyses recovered 15 and 24 modules in Bd21c in the two settings, respectively; 14 and 18 modules in Bd21d in the two settings, respectively; 14 and 16 modules in Bd3-1c in two settings, respectively; and 15 and 20 modules in Bd3-1d in the two settings, respectively. Gene membership is summarized in supplementary data set S3, Supplementary Material online.

We next addressed metrics of module preservation across our four library types, Bd21c, Bd21d, Bd3-1c, and Bd3-1d. First, we assessed the degree to which the lists of genes in a given module are conserved among our two genotypes and two environmental conditions using a test for membership overlap. We required that module overlap must be significant in both parameter settings: moderate and conservative. To examine the overlap in gene module membership among library types we used the SuperExact test (Wang et al. 2015) and a *P*-value cut-off of < 10^−12^ with the alternative hypothesis that the size intersect between two groups or among more groups is larger than expected by chance (supplementary data set S4, Supplementary Material online). The cut-off was chosen to ensure *P* < 0.05 after applying Bonferroni correction of multiple tests. Hypergraphs representing module overlap networks—here, termed “cliques” for subnetworks—were visualized using the R package igraph (Csárdi and Nepusz 2006; Csárdi et al. 2020).

Second, we tested for enrichment of transcripts exhibiting significant DGE in our coexpression modules—based on the conservative settings, above—using Fisher's exact tests (Wang et al. 2015). Significant enrichments (*P* < 0.001 uncorrected by test number) are reported in supplementary data set S5, Supplementary Material online and summarized in Table 1.

**Table 1.** Common Gene Ontology (GO) terms at the lowest level of ontology among modules in overlapping groups (based on all module versions False Discovery Rate [FDR] = 0.1) and correlated traits (based on moderate size module version, in specific library type; only *P* < 0.001 are reported, not corrected by multiple test)

Name (# of included modules)	Common GO	Correlated trait (*P* < 1e-03)	DGE enrichment (*P* < 1e-03; percentage when >75%)
Clique 1 (4)	Circadian rhythm		Bd3-1c6: E
Clique 2 (1)		glucose (1.1e-05)	E (97%),GxE
Clique 3 (2)		Bd21d13: Glucose (9.4e-08) Bd3-1d8: Glucose (1.6e-06)	Bd21d13: E (100%); Bd3-1d8: E (92%),GxE
Clique 4 (3)		Bd3-1d19: leaf water content (8.5e-04)	Bd21c13: G (76%); Bd3-1c2: E,G,GxE; Bd3-1d19: G (97%)
Clique 5 (1)	Phosphatidylethanolamine biosynthetic process		
Clique 7 (1)	Glutathione metabolic process; alternative respiration; response to chemical		GxE
Clique 8-1 (4)	Ribosomal small subunit assembly; translation; photosynthetic electron transport in photosystem II; photorespiration; ATP synthesis-coupled proton transport; reductive pentose-phosphate cycle; electron transport coupled proton transport, respiratory electron transport chain		
Clique 8-2 (4)	Electron transport chain, cellular nitrogen compound biosynthetic process, cellular respiration		
Clique 8-3 (4)	Translation		
Clique 8-4 (4)	Cytoplasmic translation; regulation of actin polymerization or depolymerization; ribosomal large subunit biogenesis, protein maturation		
Clique 8-5 (4)	Photorespiration; reductive pentose-phosphate cycle	Bd3-1d4: Leaf water content (1.5e-04) daily water usage (1.9e-04)	Bd21c2: E,G; Bd21d5: E (82%);
Clique 8-6 (4)	Response to light stimulus; photosynthesis, light harvesting in photosystem I; photosystem II stabilization; pigment biosynthetic process	Bd3-1d21: daily water usage (4.4e-05)	Bd21c2: E,G; Bd21d5: E (82%); Bd3-1c3: E; Bd3-1d21: E,G,GxE
Clique 8-7 (4)	Glycogen metabolic process		Bd21c8: E,G; Bd21d11: E,G,GxE; Bd3-1c5: E,G,GxE; Bd3-1d21: E,G,GxE
Clique 8-8 (4)		Bd3-1d12: Glucose (6.4e-05)	Bd21c8: E,G; Bd21d11: E,G,GxE; Bd3-1c5: E,G,GxE; Bd3-1d12: E,GxE
Clique 8-9 (2)	Response to wounding; phenylpropanoid biosynthetic process; regulation of defense response; monocarboxylic acid metabolic process		Bd21c5: G,GxE; Bd21d2: E,G,GxE
Clique 8-11 (3)	Plant-type primary cell wall biogenesis; plant-type secondary cell wall biogenesis; cellulose biosynthetic process		Bd21c9: E,G (100%); Bd21d6: E,G (95%); Bd3-1c10: E,G (82%)
Clique 8-12 (2)	Terpenoid biosynthetic process; chlorophyll metabolic process; pigment metabolic process	Bd3-1d21: daily water usage (4.4e-05)	Bd3-1c14: E,G (76%) Bd3-1d21: E,G,GxE

DGE enrichment in modules is shown (based on constraint module size version; only *P* < 0.001 are reported, not corrected by multiple test, and the percentage is shown if >75%). Cliques with no common GO terms or correlated traits identified are not shown. Complete lists are available in supplementary data sets S5 to S9, Supplementary Material online.

Third, biological roles of each module were inferred using Gene Ontology (GO) term enrichment, by inspecting gene annotations, and by testing for module correlation with the sampled physiological and metabolic traits. Module GO enrichment was conducted using the PANTHER classification (Thomas et al. 2003) and implemented in rbioapi (Rezwani et al. 2022), with Fisher's exact statistical overrepresentation tests of GO biological process. Only the significantly enriched GO terms at False Discovery Rate (FDR) = 0.1 and the lowest-rank GO categories are reported. Here the enrichment is reported if positive in modules of any of the parameter settings (supplementary data set S6, Supplementary Material online). Common GO terms among modules in an overlapping clique are considered to understand functions of the cliques. For comparisons of common GO term among modules in an overlapping clique, all GO categories were considered (summarized in Table 1). Common genes and annotations in overlapping cliques are presented in supplementary data sets S7 to 8, Supplementary Material online. Gene annotations were from MapMan (https://mapman.gabipd.org/mapmanstore) based on the Phytozome database, and Arabidopsis ortholog functions are searched through The Arabidopsis Information Resource (TAIR) (Rhee et al. 2003). Correlations between module eigengenes (i.e. the first principal component of each module) and traits were performed in each library type. The correlation *P*-values are reported in supplementary data set S9, Supplementary Material online and summarized in Table 1 based on moderate criteria modules, uncorrected for the number of tests.

Finally, we tested the hypothesis that genes within modules share common DNA motifs corresponding to known transcription factor binding sites. All possible 6-mer sequences were tested for enrichment in the −500 to +200 upstream window regions (Ksouri et al. 2021) relative to each gene's predicted transcriptional start site in the Bd21 reference genome, in the module using AME in MEME suite (McLeay and Bailey 2010). The AME parameters were –verbose 1 –scoring avg –method fisher –hit-lo-fraction 0.25 –evalue-report-threshold 10.0, with the control set based on all the genes. The Arabidopsis DAPSeq database (O’Malley et al. 2016) was used as a reference to interpret enriched motif sequence identified. Here the enrichment is reported for moderate criteria modules.

### Causal Network Inference in Modules

Methods such as WGCNA, based on correlations in transcript abundance, cannot provide information about the direction of regulatory interactions. Our high replication provided the power to model genes as random variables and to infer conditional independence of gene expression to represent possible causality. Here, we used the Unknown-Targeted Interventional Greedy Sparest Permutation (UT-IGSP) algorithm (Squires et al. 2020) to infer regulatory causality, representing a group of genes as a directed acyclic graph (DAG). We used all genes in a given WGCNA module—estimated from one library type—as priors to reduce latent variables in the model, relying on the simplifying assumption that there are no edges among genes between modules. Since our hypothesis of regulation was based on estimates of transcript abundance, gene “interaction” could represent either direct transcription factor-target regulation or indirect regulation arising from system-level phenomena such as metabolism or the cell cycle (Xiong et al. 2013). We included samples that the module was inferred from (here, representing “observational” data) as well as samples from all other library types (here, representing “interventional data”), representing the assumption that most of the gene regulation structure is the same between library types. In these analyses, we used the conservative module criteria (most restrictive inclusion in the WGCNA analyses) and limited the analyses to modules with the number of genes equal to fewer than 70% of samples in the specific library type. When testing for modules that are conserved within a treatment or within a genotype, modules with up to 70 genes could be used because more samples are available for inference. We used stability selection (Meinshausen and Bühlmann 2010) as implemented in Belyaeva et al. (2021) to minimize the effect of hyperparameters. Edges with a selection probability higher than 0.95 (or 0.97 for bigger conserved modules to ensure sparse edges) quantile were kept. Genes not found to be connected to other genes are not shown. DAGs were exported from R using the rgraphviz and visualized using Graphviz Visual Editor (Hansen et al. 2020).

### Identifying Patterns of Variation in Gene–Gene Interactions

We extended our analysis of gene expression responses using a regression approach that models the magnitude and direction of putative interaction between genes. For a putative regulator-target pair identified in our DAGs, we regressed the target gene transcript abundance on the regulator transcript abundance and tested whether the slope and intercept coefficients of regression changes as a function of genotype or environmental treatment. A multivariate linear regression was applied when there were multiple inferred regulators for a target gene.

For the hypothesis that gene $x_{i}\;(i=1,\;\ldots ,n)$ regulates gene *y*, to infer the intercept or slope differences between genotypes, treatment, or their interactions, we modeled their (log_2_-transformed) gene expression $x_{i}$ and *y* as:


$$y=\gamma_{0}+\gamma_{g}g+\gamma_{e}e+\gamma_{ge}g\times e+\overset{n}{\underset{i=1}{\sum }}\;(\beta_{0,i}+\beta_{g,i}g+\beta_{e,i}e+\beta_{ge,i}g\times e)\times x_{i}$$

Here g,e were used to differentiate the dataset used to train the parameters (i.e. g,e are “0,0”, “0,1”, “1,0”, and “1,1” for Bd21 control, Bd21 drought, Bd3-1 control, and Bd3-1 drought, respectively). Thus, consistent with the DGE test using DESeq2, above, a significant *P*-value of $\gamma_{g},\beta_{g}\;$ suggested a genotypic effect on regression intercept and slopes, $\gamma_{e},\beta_{e}\;$ suggest an environmental effect on intercept and slopes, and $\gamma_{ge},\beta_{ge}\;$ suggest a G × E effect of intercept and slopes. Only genes with regression *R*^2^ greater than >0.5 were considered, as estimated with the Car package in R (Fox et al. 2020). A *P*-value of <0.05 was reported to be significant without multiple test correction. Note that if the transcript abundances were standardized before fitting our regression models, the slope differentiation test should closely match the results of CILP tests introduce by Lea et al. (2019), which we included as supplementary fig. S4, Supplementary Material online.

### Allele-Specific Expression

We used allele-specific expression (ASE) to assess whether G × E in transcript abundances were caused by cis*-* or trans*-*acting variants. Bd21 served as a pollen donor on emasculated Bd3-1 flowers, resulting in ten F1 hybrid seeds. To confirm successful outcrossing in this largely selfing species, we extracted DNA from leaves (DNeasy Plant Pro Kit; Qiagen, MD, United States) and tested for a known length polymorphism on a 3% agarose gel. We performed a dry-down experiment on these F1s and extracted leaf RNA, as above. A total of 100 ng of RNA for each of ten samples along with 12 samples (three replicated of each genotype and condition) from the initial experiment were aliquoted for sequencing. For DGE, and to identify Single Nucleotide Polymorphisms (SNPs) between alleles, we generated libraries using NEB Ultra II Directional RNA with Poly(A) Selection (New England Biolabs, Ipswich, MA). Libraries were sequenced on an Illumina Novaseq generating 34.1 ± 13.7 million raw reads per sample.

To generate a reference diploid transcriptome, RNAseq reads of each Bd3-1 sample were aligned to the Bd21 reference genome (Bdistachyon_556_v3.2) based on a RNAseq short variant discovery pipeline (GATK Team 2022), with STAR alignment (Dobin et al. 2013) of two pass and alignSJoverhangMin = 30 and alignSJDBoverhangMin = 10, variants calling GATK (der Auwera and O’Connor 2020) and the flag dont-use-soft-clipped-bases. The variants called in each sample were then filtered to find SNPs and in/dels with homozygous alternative genotype mapping. To reduce false discovery, only variants identified in more than half of the replicates of each genotype were kept. The synthesized Bd21 or Bd3-1 vcf together with Bd21 genome and annotation gff was then used to generate de novo Bd21 or Bd3-1 genome using g2gtools (Kwangbom et al. 2017) and a diploid genome with emase (Raghupathy et al. 2018). The reads from all the samples were mapped to this diploid transcriptome using bowtie (Langmead et al. 2009), and a count matrix was estimated from alignments using emase (an alternate pipeline employing Bowtie2 followed by RSEM provides similar results; not shown) to account for multimapping. Isoforms were not differentiated in this study.

Genes with different counts between two alleles were considered to have sequence variants, but sparse differences in a gene might not accurately differentiate two alleles. Accordingly, we included 3963 genes that have more than >1 count of the allele in one accession and <20% total count of the allele in the opposing accession in all samples, modified from Gould et al. (2018). The RNASeq count matrix was normalized using Relative Log Expression in DESeq2, as above. All genes (>0 in more than ten samples, including allele-indifferentiable genes) were included in the normalization step with allele-distinguishable genes shown as allelic expression and other genes as total expression to ensure the normalization factors are calculated based on most genes for the comparable of genes with allelic variants. Possible read-mapping bias was assessed as in Lovell et al. (2016) shown in supplementary fig. S5, Supplementary Material online. PCA of samples are shown in supplementary fig. S6, Supplementary Material online.

We employed a pipeline based on DESeq2 as introduced by Lovell et al. (2016). Briefly, the transcript abundance of a target gene is fit into the full model:


$$\begin{array}{rl} log_{2}q_{ij} & =\beta_{0}+\beta_{T}T_{i}+\beta_{A}A_{i}+\beta_{G}G_{i}+\beta_{AG}A_{i}\times G_{i}+\beta_{TG}T_{i}\times G_{i}\\ & \;+\beta_{AT}T_{i}\times A_{i}+\beta_{AGT}A_{i}\times T_{i}\times G_{i} \end{array}$$

where $q_{ij}$ is the gene expression of sample *i* for gene *j*, $T_{i}$ is treatment (control, 0; drought, 1), $\;A_{i}$ is allele type (Bd21, 0; Bd3-1, 1), and $G_{i}$ is generation (F1, 0; Parents, 1). Accordingly, $\beta_{A}$ tests for cis*-*acting regulation; $\beta_{AG}$ tests for trans*-*acting; $\beta_{AT}$ tests for cis*-*by-environment-acting; and $\beta_{AGT\;}$ tests for trans*-*by-environment-acting. Here, the approach was also modified to perform tests in each environmental condition (as a “half model”). Each test of cis/trans was done separately in each condition because of our limited power and the possibility of low expression of the gene in samples of some conditions; cis/trans by environment interaction cannot be directly tested in this analysis. *P* < 0.1 is here reported to be significant without multiple test correction. Results were summarized in supplementary data set S10, Supplementary Material online.

## Results

### Physiological Responses to Soil Drying

We measured a series of traits daily on two inbred natural accessions as soil water content was experimentally decreased to 50% of FC (hereafter “drought”) or maintained at 85% (hereafter, “control”). These measurements suggest that both accessions began reducing whole plant water usage starting on the fourth day of the dry-down (supplementary fig. S1a, Supplementary Material online; *t*-test adjusted by Tukey; *P* = 0.048) and thereafter experienced a similar magnitude of soil drying stress, with most assayed traits exhibiting clear differences between control and drying treatments by Day 6.

Linear mixed models show that both accessions respond similarly along this dry-down, with the exception of leaf glucose and fructose contents, which showed significant increase starting on Day 5 in Bd3-1 (*z*-test, *P* < 0.0001) and Day 6 in Bd21 (*P* < 0.0001; supplementary figs. S1h and 2b, Supplementary Material online). These results suggest that the genetic background of the two accessions plays a role in shaping drought responses, revealing trait-specific sensitivity to G × E effects.

We next report the results of a highly replicated experiment (*n* = 48 to 59 for each accession in each treatment) focused on contrasts between the two accessions on Day 6 of the dry-down process. These plants constitute the sampling for the following RNA analyses. Leaf starch content decreased on Day 6 (Fig. 1g; Chisq test and *P* = 1.6e-10), as did high-degree polymerized fructans (Fig. 1h; *P* = 1.6e-7), a short-term storage carbohydrate in cool-season Pooid grasses like *Brachypodium* (Livingston et al. 2009). Sucrose shows no treatment effects in the leaf (Fig. 1e; *P* = 0.48), nor do low-degree polymerized fructans (Fig. 1f; *P* = 0.055). The degradation of starch and the catabolism of sucrose might contribute to the accumulation of glucose and fructose, both of which are strongly elevated in our Day 6 samples (Fig. 1c and d; both *P* < 2.2e-16). However, leaf glucose increases to a larger degree in Bd3-1 than in Bd21, shown as a significant G × E response (Fig. 1c; *P* = 0.0015). Overall, most carbohydrates showed genotypic differences, with Bd3-1 having higher levels. Drought also increased leaf amino acid content (Fig. 1j; *P* < 2.2e-16) and protein content (Fig. 1i; *P* = 0.00015), the latter showing a G × E effect (*P* = 0.034).

### Factors Affecting the Transcript Abundance of Individual Genes

We detected transcripts from 29,740 genes, representing 91.4% of annotated genes in the Bd21 v3.2 reference genome. PCA (Fig. 2a) showed clear separation between genotypes (PC1, 15.5% variance) and between treatments (PC2, 6.4% variance), confirming that both genotype and drought had major effects on gene expression. After filtering, 26,388 genes were included in the differential expression analysis (supplementary data set S11, Supplementary Material online). At FDR = 0.05, we found 4,725 genes expressed at higher levels and 4,087 at lower levels in Bd21 compared to Bd3-1 (genotypic effect). Similarly, 3,111 genes were upregulated and 2,832 were downregulated in response to drought (environmental effect). Among these, 3,066 genes showed both genotype and environmental effects.

In response to drought, Bd21 had 4,024 upregulated and 3,983 downregulated genes, while Bd3-1 had 3,997 up and 4,010 down (Fig. 2b). A total of 538 genes showed significant G × E effects (FDR = 0.1; Fig. 2b); 107 of these were upregulated and 66 downregulated in both genotypes but with different response strengths. Some genes responded only in one genotype: 135 up and 99 down in Bd3-1 and 39 up and 60 down in Bd21. Only 21 genes showed opposite expression changes in the two genotypes. Among the 538 G × E genes, 40 were annotated as transcription factors, including a MYB-related transcription factor with the greatest G × E fold change. GO analysis showed that G × E genes are enriched for chlorophyll and starch metabolism, amino acid processes, translation, protein folding, and stress responses (supplementary data set S12, Supplementary Material online). Together, these findings reveal that drought-induced gene expression is shaped not just by the environment or genotype, but also by their interaction.

### Variation in Gene–Gene Interaction Within Co-Expression Modules

While G × E effects on individual gene expression are well documented and evident in our dataset (Fig. 2), we sought to determine whether G × E also shapes gene regulatory patterns. One way to define G × E in gene regulation is when a regulatory interaction or set of interactions is activated or deactivated by drought in only one genotype. We used an approximation of a GRN by identifying gene coexpression modules specific to certain combinations of G × E.

**Fig. 3.**
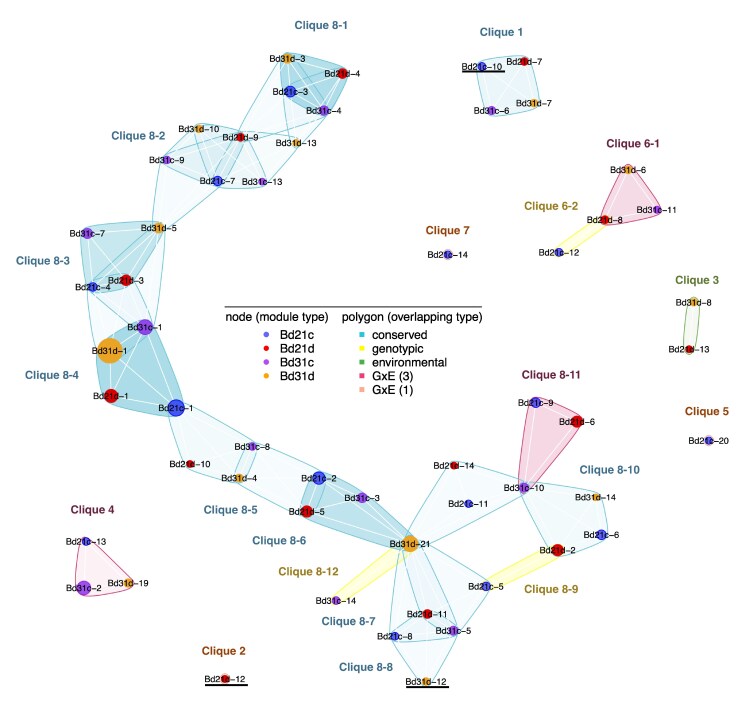
Conservation of gene coexpression modules among genotypes and treatments. Nodes represent modules identified in each of the four library types (red, Bd21d; blue, Bd21c; orange, Bd3-1d; purple, Bd3-1c), with the size of the node corresponding to the number of genes in each module. Clusters of modules enclosed by a polygon—cliques—show significant gene overlap using the high-dimensional Fisher's exact test (data are available in supplementary data set S4, Supplementary Material online). Polygon color denotes the pattern of module conservation among libraries (teal, four-module stable overlap; pink, three-module overlap; green, two-module of same environment overlap; yellow, two-module of same genotype overlap; and orange, module uniquely identified in a single library type) with the transparency of the color proportional to the number of genes in common. Modules with names underlined are shown as inferred network in Fig. 4.

The high replication used in our RNA sequencing experiment (*N* = 48 to 59, subsampled to 48 samples for each library type) affords us the power to estimate coexpression relationships independently in each of the four library types (Bd21 control watering, Bd21 drought, Bd3-1 control, and Bd3-1 drought) using WGCNA (Langfelder and Horvath 2008). We identified 14 to 24 modules per library type, depending on network parameters and library (Fig. 3). We define activation of the same module across library types as the overlapping of genes in the modules inferred from different library types. To assess overlap among modules across conditions, we used the SuperExact test (Wang et al. 2015), a high-dimensional Fisher's exact test, and then grouped significantly overlapping modules into “cliques.” These cliques were classified into four regulatory patterns: (i) **conserved** modules, found in all four conditions; (ii) **genotype-specific** modules, shared across treatments within a single genotype’ (3) **environment-specific** modules, shared between genotypes under the same condition; and (4) **G × E-specific** modules, present in three or only one conditions.

We identified 20 **conserved cliques** (Fig. 3, teal), comprising 40 modules with significant shared gene membership across the four conditions. Thirty-six of these modules showed significant GO term enrichment, including Clique 1, within which all the modules are enriched with circadian rhythm functions. Other conserved cliques are observed to be enriched for core cellular processes including the electron transport chain, cellular respiration, protein translation, photosynthesis light reactions, and glycogen metabolic process (Table 1). Some modules in conserved cliques are highly correlated with physiological traits; module Bd3-1d21 in Clique 8-6 is correlated with daily water usage (*P* = 4.4e-5) as is Bd3-1d4 in Clique 8-5 (*P* = 1.5e-4), along with tissue water content (*P* = 1.9e-4), while both modules are enriched with GO terms associated with photosynthesis. Bd3-1d12 in Clique 8-8 is correlated with leaf glucose content (*P* = 6.4e-5) and partially overlaps with Clique 8-7, which is enriched for glycogen metabolic process.

**Genotype-specific cliques** (Fig. 3, yellow) comprise modules found in only one genotype. Clique 8–9, for example, is specific to the Bd21 accession and is enriched for genes involved in wounding response and phenylpropanoid biosynthesis (including *B. distachyon* orthologs of genes encoding C4H (Bradi2g31510), PAL (Bradi5g15830), 4CL (1753) and F3H (Bradi2g54090, Bradi5g19250, and Bradi2g54090), F3′H (Bradi4g31380), PPO (Bradi2g52260), and an R2R3 MYB transcription factor (Bradi2g11676), which regulates phenylpropanoid biosynthesis in other species (supplementary data sets S7 to 8, Supplementary Material online). Clique 8–12, specific to Bd3-1, is enriched in chlorophyll and pigment metabolic processes and terpenoid biosynthetic processes; it contains five NAC regulators/domain-related genes (Bradi1g17480, Bradi1g63600, Bradi3g59380, Bradi4g02060, and Bradi4g44000) as well as a chlorophyll A–B binding protein (Bradi4g31257) and a putative senescence-inducible chloroplast stay-green protein (Bradi4g36060).

We identified **one environment-specific clique** (Clique 3, Fig. 3, green), found under drought conditions in both genotypes. Although the two modules have no GO terms in common, constituent module Bd21d13 is enriched with GO terms including response to water and contains drought-related genes including two RAB18 orthologs (Bradi1g37410 and Bradi3g43870), one rice ASR5 ortholog (Bradi4g24650), and one IAR4 (Bradi1g44480).

Finally, we identified **G × E-specific cliques** that are present in only one (Fig. 3; orange) or three (Fig. 3; pink) out of the four library types. Clique 2, for instance, is unique to Bd21 drought and includes genes enriched for a predicted bZIP transcription factor binding motif (*P* = 2.6e-7); moreover, the annotations of several genes in this module relate to abscisic acid (ABA) signaling and regulation. We also identified three cliques that are absent from one condition, including Clique 8–11, which lacks a Bd3-1 drought module despite showing enrichment of genes associated with cell wall biogenesis in the other three conditions.

Together, these results reveal that G × E effects are not limited to differential gene expression (DGE) but extend to gene coexpression networks. G × E jointly shape putative regulatory modules, indicating that drought alters transcript abundance as well as altering gene regulation in a genotype-specific manner.

### Trait Responses Support the Biological Relevance of Modules

To validate the functional relevance of the reconstructed coexpression modules, we examined whether module conservation correspond to observed trait responses; genotype-, environment-, or G × E-specific modules should align with corresponding patterns in phenotypic traits. As one example, we observe that a module related to chlorophyll metabolism is specific to Bd3-1 (Clique 8-12), and we observed a genotypic difference in relative chlorophyll content between Bd21 and Bd3-1 (supplementary fig. S1j, Supplementary Material online). Similarly, leaf glucose content showed both strong environmental and G × E effects, which matches the pattern of two of its associated modules, Clique 3 and Clique 2 (Table 1). These results suggest that trait-level variation supports underlying regulatory architecture.

### Inferring Regulatory Interactions Within Modules via Causal Inference

The preceding analyses show that some coexpression modules are conserved while others vary with genotype, environment, or their interaction (G × E). However, coexpression alone only captures correlations, in which many gene–gene interactions are likely indirect and provide no information about regulatory directionality. To infer potential causal relationships among genes within modules, we applied causal inference using the UT-IGSP algorithm (Squires et al. 2020). This approach estimates GRNs by identifying conditional dependencies between genes by inferring gene regulation distribution across our sampled replicates. The inferred networks enable us to explore changes in specific gene regulatory interactions.

**Fig. 4.**
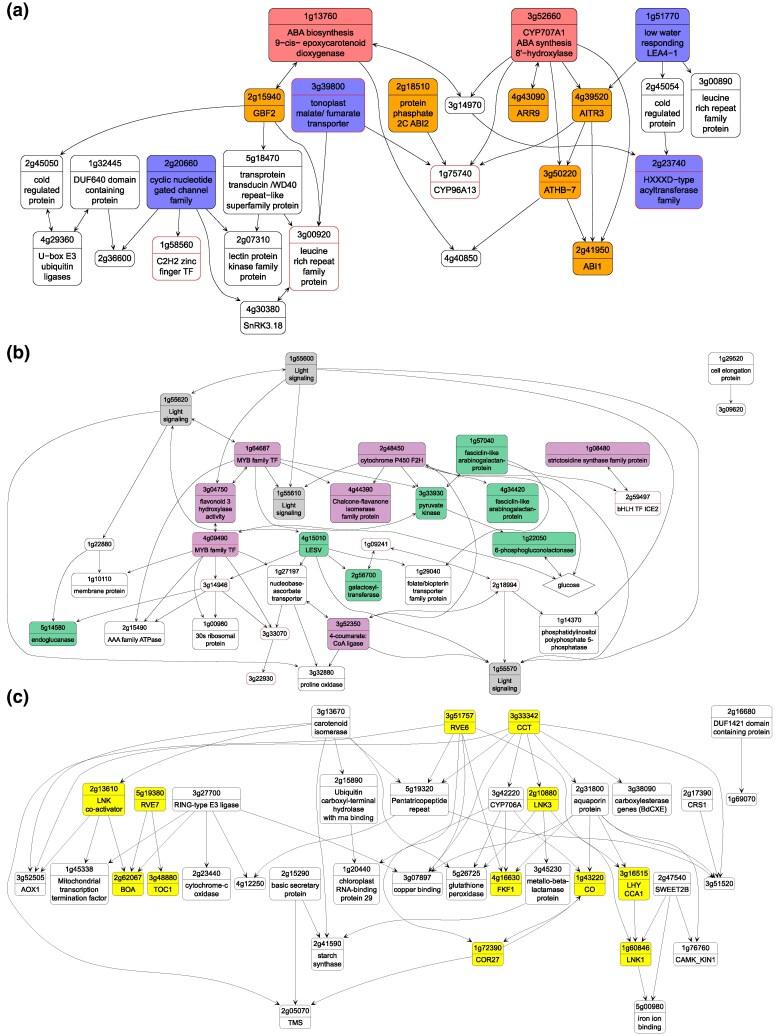
Inferred directed interactions within modules. a) Module 12 in Bd21d, b) Module 14 in Bd3-1d, and c) Module 10 in Bd21d inferred using the UTI-GSP method. Nodes are genes and edges are inferred causal interactions. Edges with two arrows indicate either that the direction of interaction is uncertain or that the interaction is inferred to be in both directions. The second row of each node shows annotations for orthologs in *Arabidopsis* or rice. Glucose is included as a node in module Bd3-1d12 as an example of a module with a significant correlation between a trait and a module eigengene (Table 1). Genes exhibiting a significant pattern of G × E in our DGE analyses are highlighted with red outline.

Bd21d12 is the sole module in Clique 2, which we showed, above, is G × E-specific. Many of the genes in Bd21d12 are annotated with functions in ABA biosynthesis or signaling. Bradi1g13760, an ortholog of AtNCED2, a 9-cis-epoxycarotenoid dioxygenase, and Bradi3g52660, orthologous to AtCYP707A1 with inferred ABA 8′-hydroxylase activity, are predicted to act upstream many other genes in the module (Fig. 4a, red fill). Bd21d12 also contains genes whose orthologs are ABA-induced or ABA-dependent regulators (Fig. 4a orange shade), including Bradi4g39520 (orthologous to a transcription repressor, AtAITR3), Bradi3g50220 (orthologous to ABA-responsive ATHB-7), Bradi2g15940 (orthologous to AtGBF2, an ABA-regulated bZIP transcription factor), and Bradi2g41950 and Bradi2g18510 (orthologous to AtHAB2 and AtHAB1, respectively). Other genes are associated with drought response in *Arabidopsis* (Fig. 4a, blue shade), including stomatal movement (Bradi2g20660 and Bradi3g39800), wax biosynthesis (Bradi2g23740), and a late embryo abundant (LEA) protein (Bradi1g51770). Notably, the gene with the strongest G × E effect in our dataset, Bradi3g00920 (Fig. 2c), has the highest eigenvalue in this module, suggesting a possible candidate gene whose expression variance drives the broader patterns in Bd21d12.

Module Bd3-1d12, part of Clique 8-8 (Fig. 4b), is correlated with leaf glucose content. Bradi1g55600, inferred to act upstream in the module, is an early light-induced protein (ELIP2 ortholog) involved in chlorophyll synthesis, with several paralogs also present in the module (Fig. 4b, gray filling). Downstream genes include genes involved in primary metabolism (Fig. 4b, green shade), such as Bradi3g33930 (pyruvate kinase), Bradi1g22050 (6-phosphogluconolactonase), Bradi5g14580 (endoglucanase), Bradi2g56700 (galactosyltransferase), and Bradi1g18650 (glucanotransferase), as well as genes involved in starch metabolism (e.g. Bradi4g15010 and LESV ortholog). When we include glucose as a node in the module, we find that it is predicted to directly interact with three genes including Bradi1g22050, the 6-phosphogluconolactonase. The module also contains genes with predicted functions in secondary metabolism (Fig. 4b, purple fill), including orthologs of an *Arabidopsis* MYB family transcription factor involved in flavonol biosynthesis (Bradi1g64687), flavonoid 3′-hydroxylase (Bradi3g04750), flavanone 2-hydroxylase (Bradi2g48450), and a 4-coumarate:CoA ligase in the phenylpropanoid pathway (Bradi3g52350). Phenylpropanoids and flavonoid derivatives are generated in *B. distachyon* (Piasecka et al. 2022). Although no studies directly tested the increase of these compounds as a defense mechanism, the knockdown of PAL1 lowered levels of cinnamic acid, salicylic acid, lignin precursors, and compromised resistance to *Panicum mosaic virus* and satellite virus (Pant et al. 2021). Notably, several unannotated genes are connected in the module and show G × E in expression suggesting novel pathways involved in G × E gene expression and possible information passing along the network.

Finally, we discuss Clique 1 as an example of a clique that is conserved in all four library types. We investigated one constituent module, Bd21d10, as a representative of the clique and find that it is enriched in genes relate to circadian processes (Fig. 4c, yellow shade). There are five predicted MYB TFs in Bd21d10, including Bradi3g51757 (orthologous to AtREVEILLE 6), Bradi2g62067 (orthologous to AtBROTHER OF LUX ARRHYTHMO and OsLUX ARRHYTHMO), Bradi5g19380 and Bradi1g29680 (orthologous to AtREVEILLE 2/7), and Bradi3g16515 (orthologous to AtLATE ELONGATED HYPOCOTYL 1, and OsCIRCADIAN CLOCK ASSOCIATED 1). Additional genes in this module are orthologous to CONSTANS (CO) and related proteins in *Arabidopsis*. Genes in circadian-controlled pathways show conserved oscillation over 24 h intervals (Filichkin et al. 2011); we hypothesize that stochastic clock variance among our sampled individuals may have provided the signal for reconstructing these conserved interactions in our data.

Here, causal inference allowed us to propose putative regulatory relationships on which we will next explore the possible effects of G × E. The predicted GRNs are consistent with known biological functions, providing a mechanistic basis for understanding how G × E jointly shape regulatory networks.

### Genetic and Environmental Effects on the Interactions Among Genes

An edge between nodes in our inferred GRN represents a putative directional interaction between two genes. Having previously identified G × E effects at the module level, we next asked whether such effects influence regulatory interactions between genes. The high biological replication of our RNASequencing across four library types enables us to fit linear models to explore the effects of genotype, environment, and their interaction on the slope and intercept of the regulation affecting an expression relationship between pairs of genes. We identified seven possible cases.

**Fig. 5.**
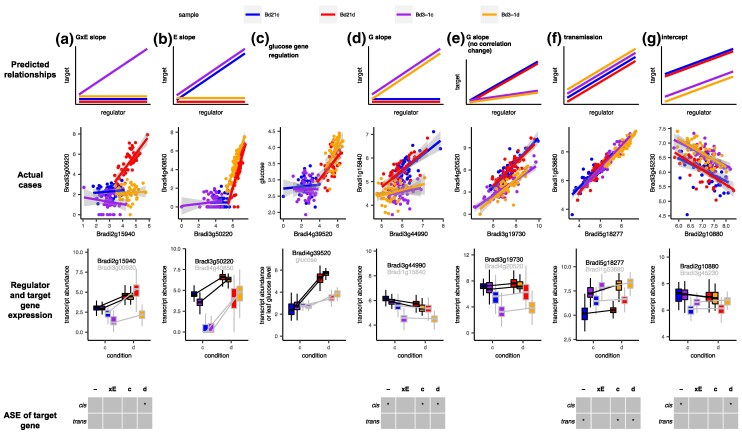
Predicted and observed examples of regulation between pairs of selected genes. a, b, and d to g) Regulation slope changes, transmission, and regulation intercept changes. These are illustrated with linear regression between regulator and target, the expression level of each gene, and ASE patterns. ASE patterns include constitutive regulation (-) and environment specific (xE) detected in full model (see Materials and Methods), or regulation detected in control (c) and drought (d) condition. *P* < 0.1 and not corrected for multiple tests. c) Regression of log-transformed leaf glucose content on upstream identified gene Bradi4g39520 as a putative regulator of leaf glucose content. Transcript abundances are log2 (norm_count + 1).

First, **regulatory slopes can vary by environment**. Some gene pairs show significant changes in slope between conditions, suggesting environment-dependent regulation (Fig. 5b). For example, in module Bd21d12—unique to Bd21 under drought—the relationship between Bradi4g40850 (unknown function) and Bradi3g50220 (an ABA-responsive transcription factor, ATHB-7 ortholog) is significant only under drought. Similarly, in module Bd21d13, Bradi1g44480 (IAR4 ortholog) is inferred to regulate Bradi1g37410 (a dehydrin) only under drought (supplementary fig. S7a, Supplementary Material online). Across modules, we see clustering of context-dependent edges, such as module Bd21d13 in Clique 3—reconstructed only under drought conditions—comprising 32 edges, of which 14 (44%) are environmentally dependent (supplementary table S2, Supplementary Material online).

Second, **regulatory slopes can vary by genotype** (Fig. 5d). For example, in Bd21-specific module Bd21c5, Bradi3g44990 (a putative cell-wall invertase) is predicted to regulate Bradi1g15840 (putative allene oxide cyclase in jasmonic acid synthesis) only in Bd21. Consistent with the observation that the genes in Bd21c5 are only coexpressed in Bd21, we find that Bd21c5 has 12 edges showing a significant effect of genotype among its among 65 edges (18%; supplementary table S2, Supplementary Material online).

Third, we observe **G × E effects on regulatory slopes**—where interaction is significant in just one of the four library types (Fig. 5a). For instance, in Bd21d12, discussed above and enriched for ABA-responsive genes, Bradi2g15940 (GBF2 ortholog) shows a G × E-specific interaction with Bradi3g00920 (leucine-rich repeat family protein), a gene with strong G × E in expression.

Fourth, **regulatory interactions can vary in strength but with conserved direction**. In some gene pairs, the regulatory relationship is present across all conditions, but the strength of the interaction (slope) differs. For example, Bradi4g20520 (unknown function) regulates Bradi3g19730 (jacalin-like lectin domain-containing protein) in all conditions, but the slope is higher in Bd21 than Bd3-1 (Fig. 5e), possibly due to stronger binding affinity in Bd21.

Fifth, **baseline expression (intercept effects) can be different**. Some relationships show consistent slopes but varying baseline expression across library types (Fig. 5g) possibly due to the presence of cofactors or the general resource and energy availability of the cell. In Bd21c10, Bradi2g10880 (LNK3 ortholog) regulates Bradi3g45230 (RNA-metabolizing metallo-beta-lactamase), with Bd3-1 showing a higher baseline expression, leading to genotype-dependent downstream expression. Similar intercept differences linked to genotype, environment, or G × E are widespread (supplementary table S2, Supplementary Material online).

Sixth, **regulatory interactions can be consistent across library types**. In some cases, both genes in a pair show G × E in expression, but there is no significant change in slope or intercept between them—suggesting shared regulatory transmission from an upstream factor. For instance, in Bd3-1c16, Bradi5g18277 (predicted serine-type endopeptidase) shows a G × E effect and regulates Bradi1g53680 (a predicted zinc transporter). Bradi1g53680 expression closely mirrors that of its regulator (Fig. 5f; supplementary file S1, Supplementary Material online), and their interaction remains stable across all conditions (Fig. 5f). We also found that in module Bd3-1d12, G × E genes are more likely to be regulated by other G × E genes (*t*-test, *P* = 0.013; see supplementary fig. S7b, Supplementary Material online), suggesting structured propagation of regulatory variation.

Finally, **the above patterns can be observed between genes and molecular traits**. We use our regression approach to study the transcriptional control of leaf glucose content, which shows a significant G × E effect in our metabolic assays (Fig. 1h) and is highly correlated with four module eigengenes (modules Bd21d12, Bd21d13, Bd3-1d8, and Bd3-1d12; Table 1). Nine genes are inferred to regulate glucose when it is included as a node in these modules. Among these, we find that Bradi4g39520 only exhibits an interaction with glucose under drought stress (Fig. 5c); in control conditions, the glucose content is the same between the genotypes and is not dependent on the expression level of Bradi4g39520. Bradi4g39520 is an ortholog of AITR3, an ABA-induced transcription repressor that acts as feedback regulator in ABA signaling in *Arabidopsis*.

These results provide evidence of G × E in gene–gene interactions, extending beyond individual gene expression. Furthermore, we identified diverse patterns—environment- and genotype-specific regulation, slope and baseline variation, and regulatory transmission—all of which contribute to the complex architecture of GRNs.

### ASE Reveals Cases of Cis-Regulation Contributing to G × E Regulation

We hypothesized that some of the observed regulatory differences are driven by cis- or trans-acting genetic variation, potentially reflecting evolutionary divergence between the two studied accessions. We used ASE analysis to test whether observed genotype- or G × E-associated regulatory differences were due to cis or trans effects (Fig. 5; supplementary fig. S8, Supplementary Material online). Limited by the high genetic similarity between the accessions, we can only detect regulation mechanisms in a subset of genes. In the case of the G × E-specific interaction between Bradi2g15940 and Bradi3g00920 (Fig. 5a), we found that Bradi3g00920 is under cis-regulation specifically under drought (supplementary fig. S8a, Supplementary Material online). In contrast, for Bradi5g18277 and Bradi1g53680 (Fig. 5f), which show stable interactions, ASE analysis suggests that Bradi1g53680 is controlled by a trans-acting variant (supplementary fig. S8c, Supplementary Material online) consistent with the hypothesis of transmission. Thus, the results highlight a case where G × E effects in gene regulation arise from cis-acting mechanisms, highlighting the complex genetic architecture underlying regulatory network rewiring.

## Discussion

Gene expression has long been a focus of both evolutionary and functional genetic studies. Here, we aimed to integrate these two perspectives by studying the environmental and genetic control of regulatory interactions between genes using highly replicated RNASequencing libraries of two *B. distachyon* accessions exposed to soil drying and control conditions.

We extend past work that showed that the expression of individual genes can vary as a function of genotype and environmental treatment to demonstrate variation in interactions between genes. One example highlighted throughout the analyses in this study is Bradi3g00920, which shows a pattern of G × E in its transcript abundance. By inferring causality for the module in which the gene is located, we infer that Bradi3g00920 is relatively downstream of several other genes putatively regulating it. Multiple regression of its gene expression suggests that Bradi3g00920 is regulated by Bradi2g19540 only under drought in Bd21. Further, ASE reveals that this gene is associated with a cis-regulated expression pattern.

We also exploit recently developed tools from causal inference (Squires et al. 2020) to highlight a phenomenon we term “expression transmission” to identify candidate regulatory genes apparently passing their expression pattern to downstream targets. For example, Bradi1g53680 consistently depends on Bradi5g18277 and is trans-regulated (Fig. 5f), suggesting that the causal variant lies in Bradi5g18277 or an upstream regulator. This implies that trans-QTLs may reflect regulatory cascades rather than direct changes in protein-coding sequences and also emphasizes the need to consider gene expression within a network context, rather than treating genes as independent variables.

A second key implication of our work is that variation in gene–gene interactions can lead to a coexpression module not being reconstructed in certain combinations of G × E. For example, 44% of the inferred edges in module Bd21d13 exhibit environmentally dependent regulation, and, perhaps as a result, this module is found only under drought stress. In contrast, modules that have fewer pairwise gene–gene regulatory changes—or for which gene expression states are transmitted faithfully from upstream genes in the network—may be more likely observed under all sampled conditions.

Genetic variants affecting expression, whether constitutive differences between varieties (in our study, those with an only significant G effect) or G × E, may be acted on by selection if they ultimately have an effect on phenotype. The network position of such variants matters: Highly pleiotropic regulators may have broad effects, while variants clustered within modules may limit trade-offs (Wagner and Zhang 2011). Recent work considered the network topology or pleiotropy of gene expression in the context of the evolution of plasticity (Des Marais et al. 2017a; Laitinen and Nikoloski 2019). However, these approaches may be limited by spatial separation between genetic variants and their effects on gene expression (Marchand et al. 2014; Liu et al. 2019), or between environmental sensing and transcriptional responses (Chevin et al. 2022). As a result, pleiotropic impacts as well as variation in network structure are often overlooked. We specifically consider such variation in regulatory interaction here. Our large gene expression dataset also allowed us to estimate gene coexpression independently in each combination of genotype and environmental treatment, thus relaxing the assumption of an invariant network topology across conditions used previously (Des Marais et al. 2017a; Laitinen and Nikoloski 2019; Hämälä et al. 2020a). We note that other approaches to account for network structure indirectly, such as mapping expression covariance or principle components as traits in QTL analysis (Josephs et al. 2015; Lea et al. 2019; Veltsos and Kelly 2024), may detect similar regulatory interactions.

We observed that changes in gene–gene interactions are found to be clustered in some modules, suggesting a spatial pattern of regulatory variation in networks and supporting the hypothesis that modularity allows for reduced pleiotropic effects of mutations (Wagner and Zhang 2011). A related hypothesis is that certain regulatory pathways are under weaker purifying selection, thus leading to accumulation of genetic variation in a subset of modules (Hämälä et al. 2020b). Consistent with this latter hypothesis, we observe that variable interactions are more common in modules not enriched for GO terms associated with core biological pathways such as translation and ATP synthesis processes. The small overlap between variable modules and the chain structure in Fig. 3 is representative of these patterns. Previously, we observed a similar pattern in which individual genes exhibiting expression G × E were localized at the periphery of a gene coexpression network under drought, again consistent with the hypothesis that such variants minimize pleiotropic effects between environments (Des Marais et al. 2017a). The pattern we observe here is similar, here at the level of gene–gene regulatory interactions.

While we have highlighted a number of cases where the interactions among genes vary, we do observe several modules that, while conserved among library types, are enriched for genes that respond to stress (e.g. Clique 8-6 with GO terms of light reactions of photosynthesis-related genes). These networks may remain topologically stable while still enable environmental responsiveness. The mechanism of this response—whether via active regulation or more systems-level metabolic differences—cannot be determined with this type of data, but we highlight the distinct interpretation of this pattern from modules with both environmentally specific expression and topological variation that we observe in, e.g. Clique 3 (Fig. 3).

Our study also connects cellular and whole-plant traits to specific gene regulatory features. First, modules either associated with traits or enriched with GO terms represent hypotheses about the transcriptional control of key biological responses. Notably, even in modules representing housekeeping pathways, fewer than 50% of genes have annotations; our network-based approach therefore represents a novel means to develop hypotheses of gene function. For example, nine genes among 26 genes in the intersect of Clique 1 modules are not annotated and might represent candidates for circadian rhythm studies. Second, certain traits, such as leaf glucose content, might be controlled by several pathways, as we found glucose to be associated with four modules (i.e. three modules associated with glycogen biosynthesis processes, two drought-specific modules, and the module enriched with ABA processes; Table 1). We also show how these associations may change in different environments as some of the glucose-associated modules only exist under drought stress. For example, Bradi4g39520, previously identified to be an ortholog of AITR3, may have an unappreciated role related to glucose signaling or metabolism. Moreover, the example we highlighted being G × E regulated, Bradi3g00920, is uncharacterized and may warrant further study. Collectively, these results suggest that our approach represents an unbiased and systematic way of developing hypotheses about the genes and pathways underlying biological processes.

Our framework and methodology can be extended to compare and understand gene regulation across tissues, cell types, treatments, and species (Kajala et al. 2021), (GTEx Consortium 2015; Huang et al. 2018). It especially takes advantage of highly replicated data, which is increasingly available due to rapid and affordable sequencing technologies such as single-cell transcriptomics (Shahan et al. 2022; Nolan et al. 2023; Wang et al. 2024). Incorporating experimentally validated gene regulatory interactions, e.g. from DNA Affinity Purification Sequencing (Bartlett et al. 2017), as prior knowledge in future studies can enhance the accuracy of network inferences.

Collectively, our work highlights how evolutionary processes may modify GRNs and sheds light on both understanding plasticity and engineering stress resilience. First, a gene's expression can be modified at various steps of its regulation and must be considered in a network context. Conversely, modification of a gene's expression to respond to environmental factors will likely require accounting for gene–gene interactions; our study shows one way to identify such targets. Second, regulatory variation seems to cluster in specific modules. Variants whose effects are restricted to such modules may have fewer pleiotropic effects and thereby be more likely to be retained in populations.

## Supplementary Material

msaf218_Supplementary_Data

## Data Availability

The datasets and computer code produced in this study are available in the following databases: RNA-Seq data: Gene Expression Omnibus GSE267880 (https://www.ncbi.nlm.nih.gov/geo/query/acc.cgi?acc = GSE267880) RNA-Seq data for allele-specific expression: Gene Expression Omnibus GSE267878 (https://www.ncbi.nlm.nih.gov/geo/query/acc.cgi?acc = GSE267878) Analysis scripts: GitHub (https://github.com/jyjiey/GxE_in_gene_regulation_network).
