# Supplementary material for: Genotype by Environment Interactions in Gene Regulation Underlie the Response to Soil Drying in the Model Grass Brachypodium distachyon
Source: Mol Biol Evol. 2025 Sep 11;42(10):msaf218. doi: 10.1093/molbev/msaf218 (PMC12509060; doi:10.1093/molbev/msaf218)
Supplement: msaf218_Supplementary_Data [file msaf218_supplementary_data.zip › manuscript_gxe_gene_regulation_SUPPLEMENTARY_v7.pdf]

SUPPLEMENTARY INFORMATION

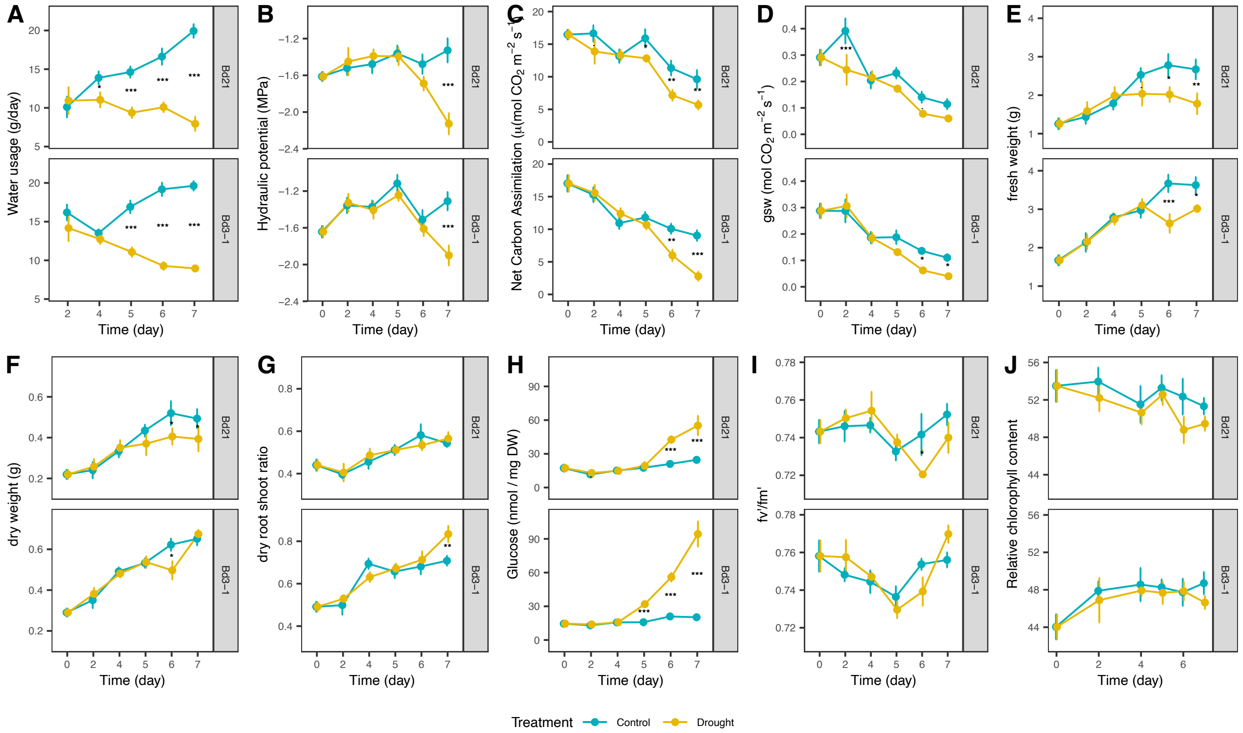

Supplementary Figure 1. Comparison of physiological and metabolic traits between control and dry-down in two accessions from Day 0 to Day 7. (Generalized) Linear models were determined based on AIC score. Random-effects components of the models were based on likelihood ratio tests. Differences of means shown here with asterisks, were determined using contrasts between the two treatments at each time point in each genotype by Tukey's test. N = 9 for each point.

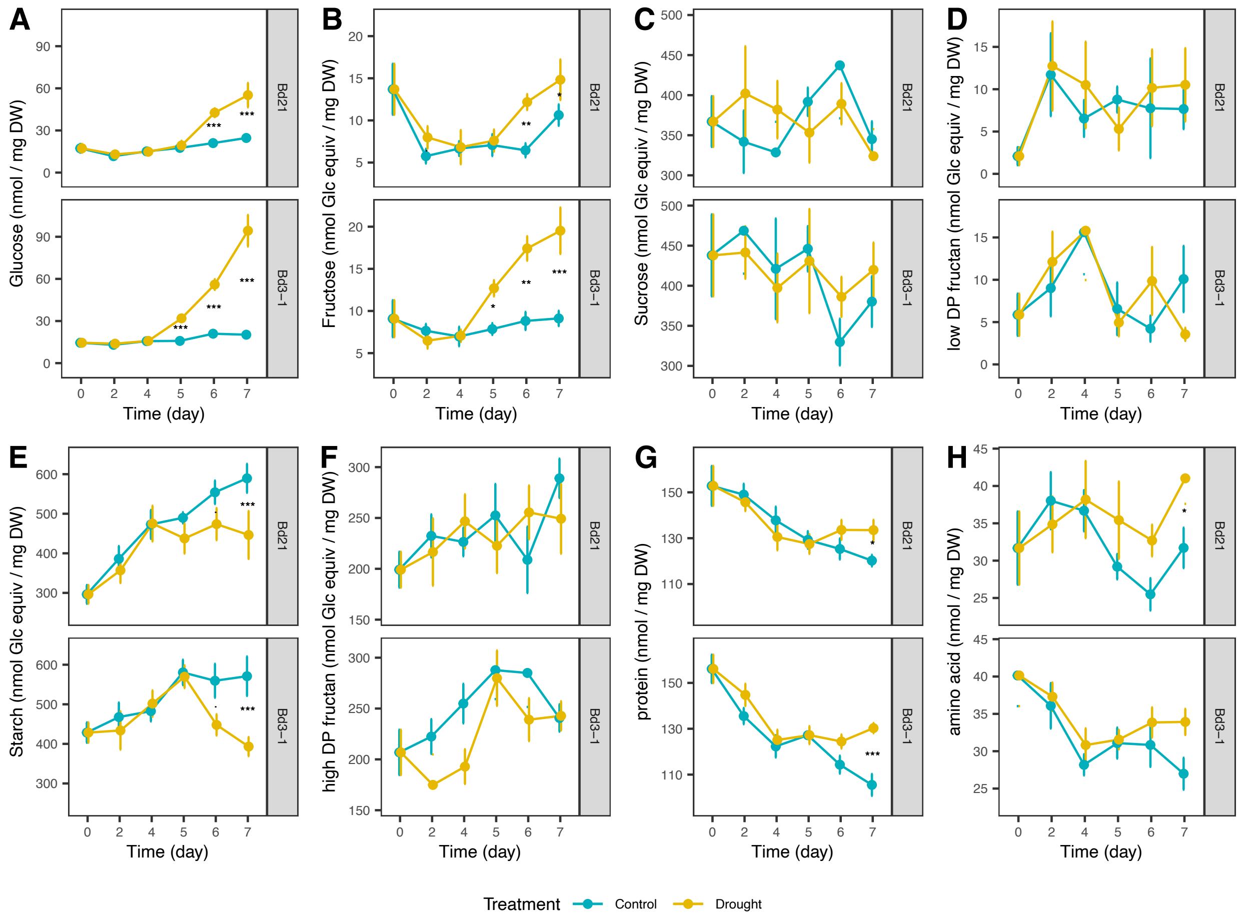

Supplementary Figure 2. Other physiological traits during dry-down in addition to Supplementary Figure 1. Comparison of physiological and metabolic traits between control and dry-down in two accessions from Day 0 to Day 7. (Generalized) Linear models were determined based on AIC score. Random-effects components of the models were based on likelihood ratio tests. Differences of means shown here with asterisks, were determined using contrasts between the two treatments at each time point in each genotype by Tukey’s test. N = 9 for each point.

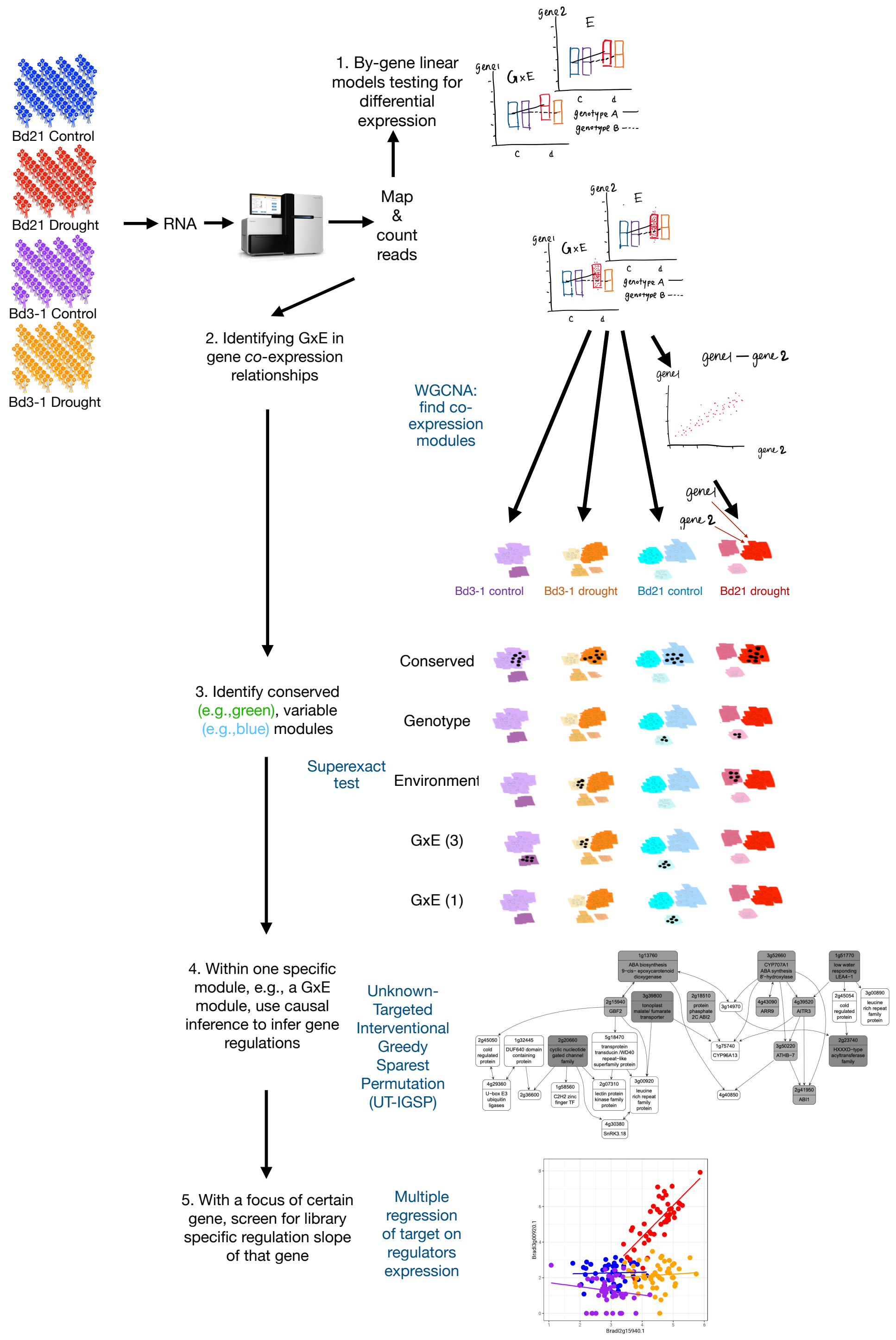

Supplementary Figure 3. Pipeline of gene expression analysis.

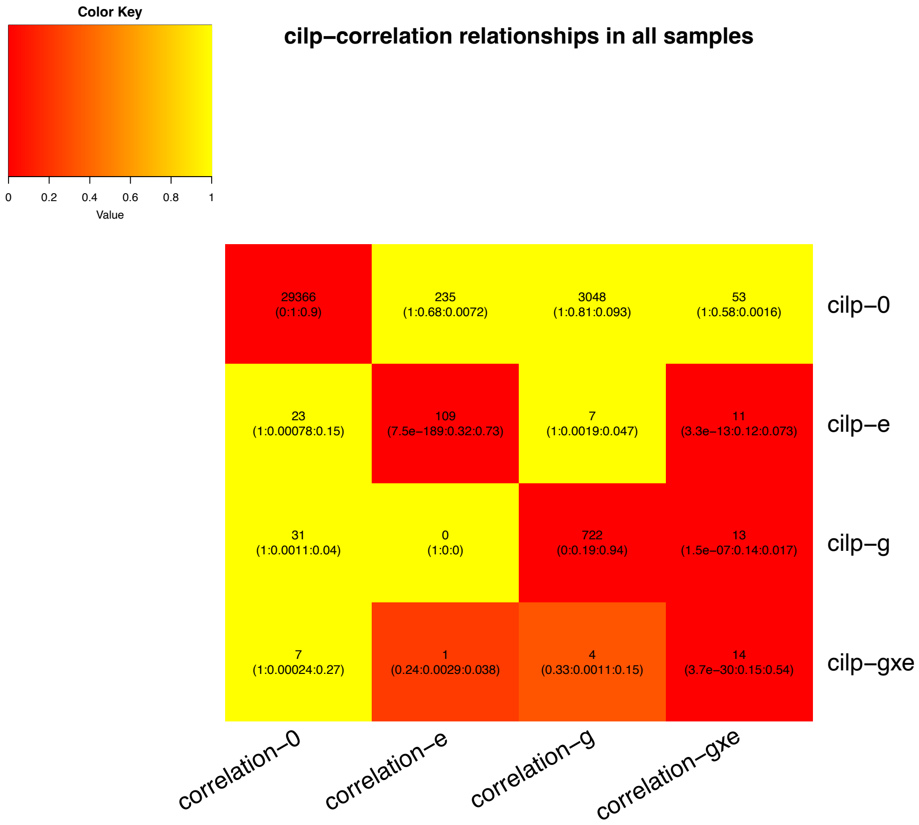

Supplementary Figure 4. Heatmap comparing CILP and correlation variation from regression method based on gene pairs with correlation >0.75 in any of the library types. The number represent the # of the cases found in the intersect. The three number in the parenthesis are p-value of fisher's exact test, the proportion of the genes in the intersect in the correlation category, and the proportion of the genes in the intersect in the CILP category. The color is based on p-value.

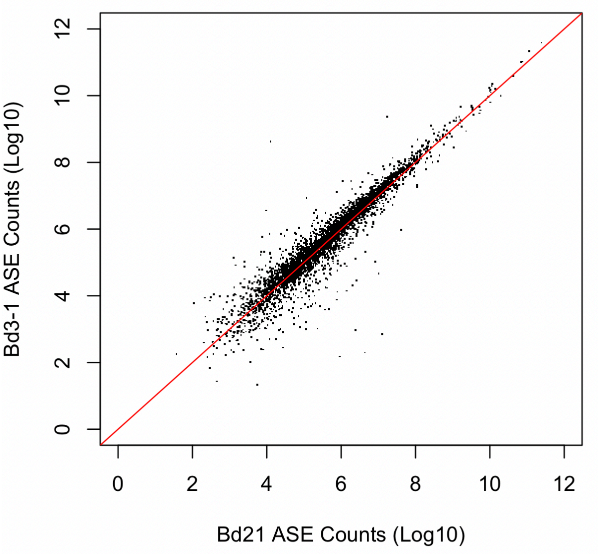

Supplementary Figure 5. mean reads of Bd21 allelic count vs. mean reads of Bd3-1 allelic count across all samples of allele specific genes suggest no alignment bias to Bd21 or Bd3-1

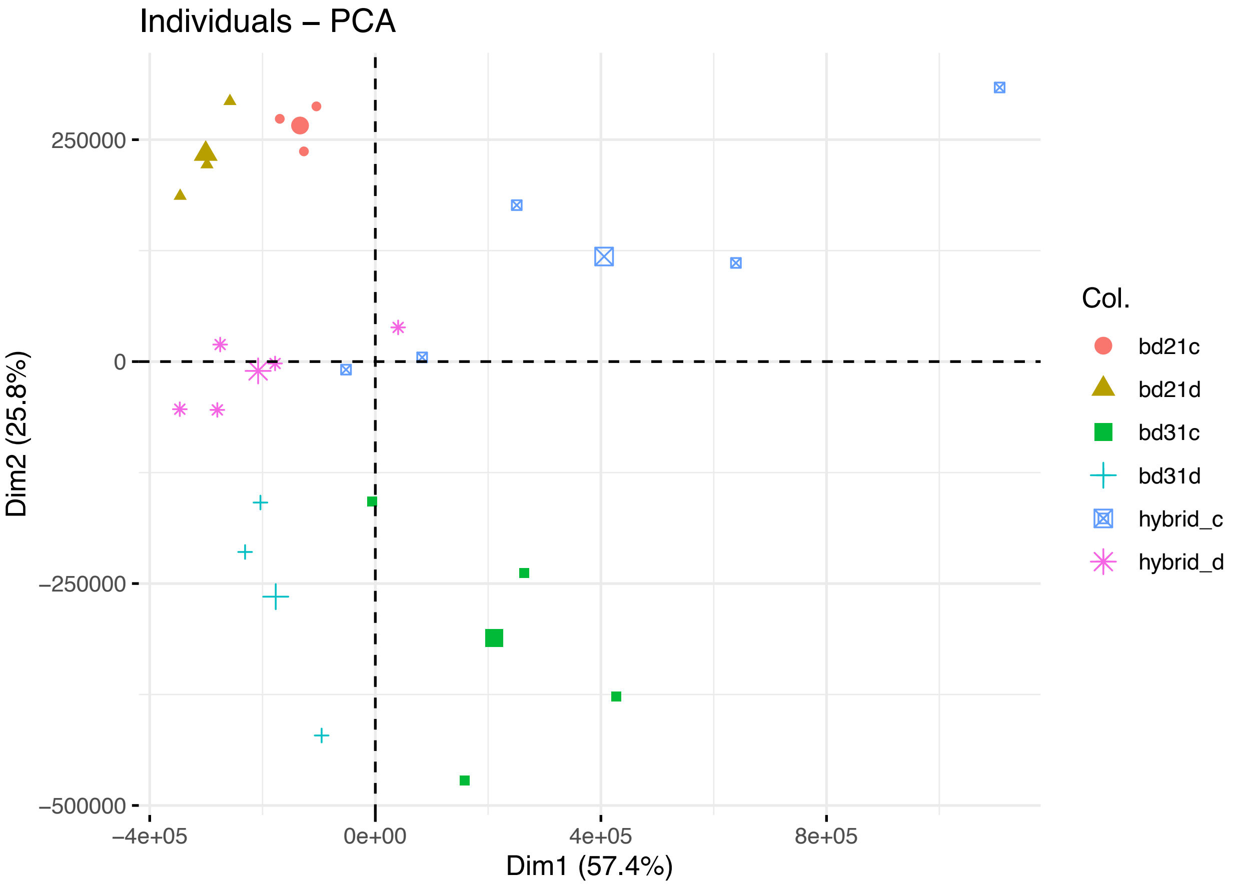

Supplementary Figure 6. PCA of allelic gene counts colored based on samples genotype and treatment (after normalization)

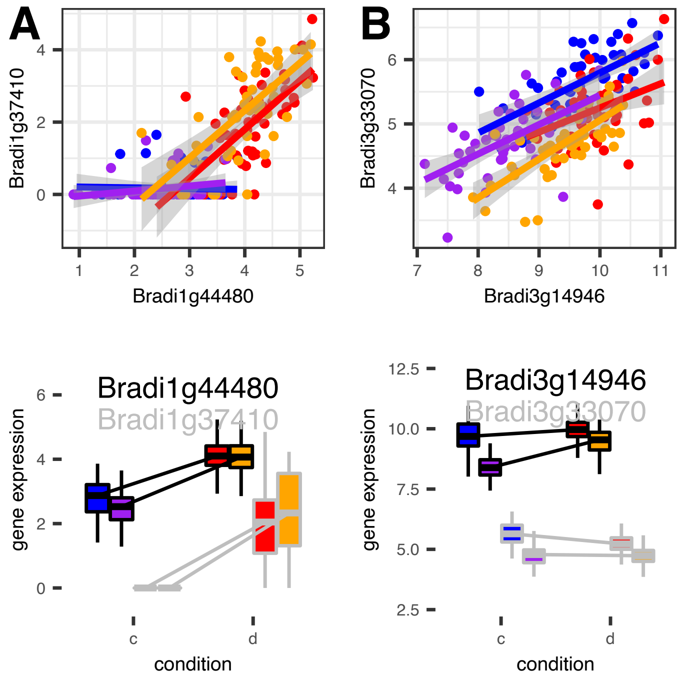

Supplementary Figure 7. Examples of regulations between pairs of genes selected in addition to Fig.6 **A)** shows regulation E slope changes, and **B)** shows transmission of GxE DGE. They are illustrated with linear regression between regulator and target, their expression levels based on log2(norm_count+1). Bd21c, Bd21d, Bd3-1c, Bd3-1d are colored with blue, red, purple, orange, respectively.

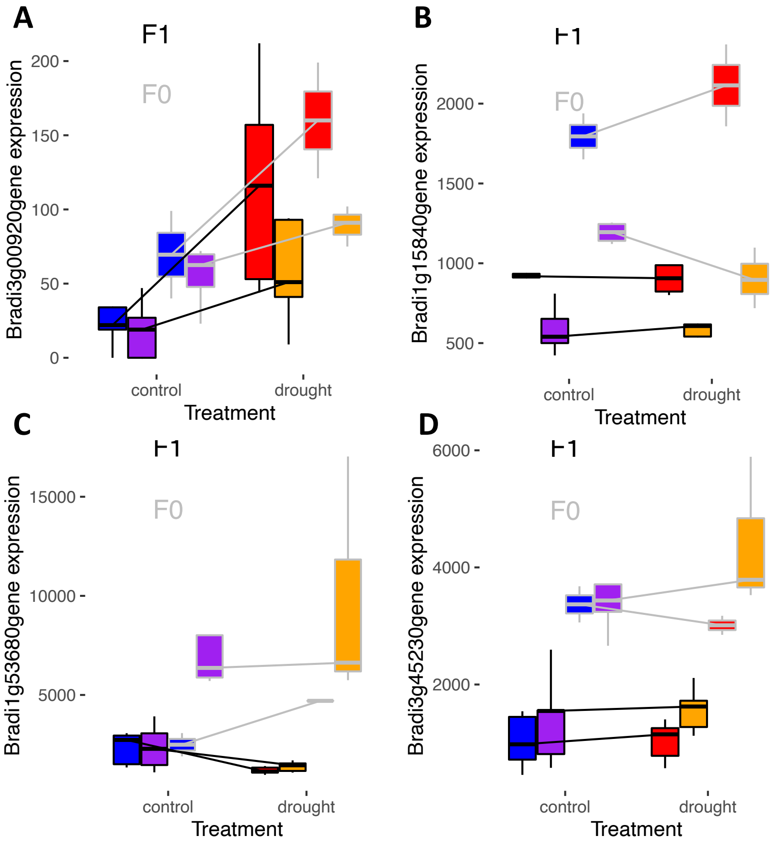

Supplementary Figure 8. ASE of the genes in Figure 6 (Bradi3g00920, Bradi1g15840, Bradi1g53680, Bradi3g45230). F1 is expression in hybrid and F0 is expression in parents. Values are normalized count. Bd21c, Bd21d, Bd3-1c, Bd3-1d are colored with blue, red, purple, orange, respectively.

Supplementary Table 1. Models used for physiological traits during dry-down reported in both Supplementary Figure 1 and 2. (Generalized) Linear models were determined based on AIC score. Random-effects components of the models were based on likelihood ratio tests.

No.	Trait	Model selected
1	Water usage	Accession +Treatment+harvest.day+ Accession*harvest.day+ Treatment*harvest.day
2	Hydraulic potential	Accession +Treatment+harvest.day+ Treatment*harvest.day
3	Net photosynthesis rate	Accession +Treatment+harvest.day+ Treatment*harvest.day

4	gsw	Treatment+harvest.day+ Treatment*harvest.day
5	Fresh weight	Accession +Treatment+harvest.day+ Treatment*harvest.day
6	Dry weight	Accession +Treatment+harvest.day+ Treatment*harvest.day
7	Dry root shoot ratio	Accession +Treatment+harvest.day+ Treatment*harvest.day
8	Glucose	Accession + Treatment +harvest.day+ Accession* Treatment +Treatment*harvest.day
9	fv'/fm'	Accession
10	Relative chlorophyll content	Accession +harvest.day
11	Fructose	Accession + Treatment +harvest.day+ Accession* harvest.day + Treatment*harvest.day+(1| DW.Plate)
12	Sucrose	Accession
13	Low DP fructan	-
14	Starch	Accession + Treatment +harvest.day+ Accession* harvest.day + Treatment*harvest.day+(1| DW.Plate..)
15	High DP fructan	harvest.day
16	Protein	Accession + Treatment +harvest.day+Treatment*harvest.day
17	Amino acid	Treatment +harvest.day+Treatment*harvest.day

Supplementary Table 2. Percentage of E, G, G+E and GxE DGE change, and slope change identified in edges or intercept change in genes in small module (<40 genes) inferred gene regulation network (e.g., Figure 4).

		edge DGE change				edge rewiring %					gene intercept change %				
module no.	total edges	G	E	G+E	GxE	G	E	G+E	GxE	total genes	E	G	GxE	G+E	Non
bd21c_13	67	25	4	10	6	10%	12%	3%	9%	33	3%	24%	24%	6%	39%
bd21c_14	52	19	8	5	0	8%	17%	0%	6%	32	6%	25%	13%	9%	28%
bd21c_17	37	16	4	6	0	5%	5%	3%	5%	27	11%	11%	15%	19%	33%
bd21c_21	30	8	4	12	0	10%	20%	3%	7%	19	5%	5%	21%	21%	37%
bd21c_22 (part of 9)	22	5	3	3	0	5%	23%	9%	5%	18	6%	22%	17%	0%	28%
bd21c_23 (part of 10)	25	5	3	10	0	4%	8%	4%	0%	18	6%	6%	17%	22%	33%
bd21d_12	41	5	11	12	11	34%	15%	5%	12%	29	3%	17%	17%	7%	31%
bd21d_13	32	2	11	7	5	44%	3%	6%	6%	25	0%	4%	32%	8%	16%
bd21d_16 (part of 5)	23	6	9	6	1	17%	4%	4%	17%	20	5%	15%	5%	25%	30%
bd31d_12	68	15	16	11	6	10%	9%	0%	1%	37	8%	8%	27%	24%	11%
bd31d_14	51	17	3	8	0	10%	4%	4%	4%	28	0%	25%	14%	21%	25%
bd31d_18 (part of 21)	32	4	10	10	2	13%	19%	9%	9%	19	11%	5%	16%	21%	37%
bd31d_19	108	44	15	18	4	7%	24%	5%	6%	33	6%	12%	15%	21%	39%
bd31c_13	79	8	4	0	0	8%	5%	3%	13%	31	19%	10%	10%	10%	48%
bd21c_5 (intersect with Bd21d_2)	65	25	9	8	0	5%	18%	2%	9%	36	14%	8%	8%	17%	39%
bd31c_10	55	11	19	10	0	7%	9%	2%	11%	33	12%	15%	21%	27%	21%
bd31c_16	27	13	1	3	0	15%	19%	0%	15%	15	7%	20%	7%	27%	33%

Supplementary File 1 Direct test of DGE change between two genes

Direct test of DGE change between two genes

We fit pairs of genes in regressions to formally test whether DGE changes occur between them. To be specific, a vector of log2 -transformed transcript abundance for two correlated genes were fit with the following model:

$$x = gene\ * g * e$$

where x is a combined vector of gene expression of two genes, and variable $gene$ $(1,2)$ differentiates which the gene of gene expression value x. Thus, the regression coefficients of $gene * g$ which differ significantly from 0 suggest that effects of genotype on transcript abundances differ between the two genes, and similarly coefficients of $gene * e$ which differ significantly from 0 suggest that effects of treatment environment on transcript abundances differ between the two genes, and significant coefficients for $gene * g\ * e$ suggests a GxE effect on transcript abundance. P-values <0.001 is reported to be significant without multiple test corrections. Results are shown in Supplementary Figure 2. This method avoids the indirect comparison by DGEs of two genes, but generally consistent with it (see Supplementary Figure 3).

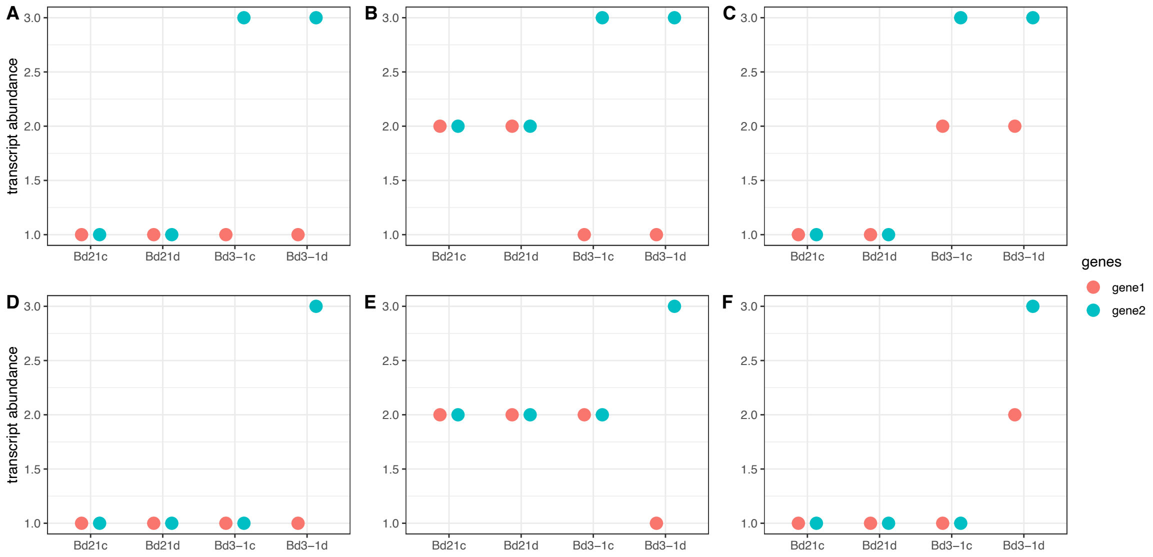

Examples illustrating gene-gene interaction. **A-C)** DGE G changes; **E-G)** DGE GxE changes. In each type of variation, the three plots show examples of different responses (**A,D**), direction (**B,E**), and sensitivity(**C,F**), respectively. Red and blue dots show two gene expression levels respectively, in four library types.
